# High internal noise and poor external noise filtering characterize perception in autism spectrum disorder

**DOI:** 10.1038/s41598-017-17676-5

**Published:** 2017-12-14

**Authors:** Woon Ju Park, Kimberly B. Schauder, Ruyuan Zhang, Loisa Bennetto, Duje Tadin

**Affiliations:** 1 Department of Brain and Cognitive Sciences, University of Rochester, Rochester, NY 14627 USA; 2 Center for Visual Science, University of Rochester, Rochester, NY 14627 USA; 3 Department of Clinical and Social Sciences in Psychology, University of Rochester, Rochester, NY 14627 USA; 4 Center for Magnetic Resonance Research, Department of Radiology, University of Minnesota at Twin Cities, Minneapolis, MN 55455 USA; 5 Department of Ophthalmology, University of Rochester School of Medicine, Rochester, NY 14642 USA

## Abstract

An emerging hypothesis postulates that internal noise is a key factor influencing perceptual abilities in autism spectrum disorder (ASD). Given fundamental and inescapable effects of noise on nearly all aspects of neural processing, this could be a critical abnormality with broad implications for perception, behavior, and cognition. However, this proposal has been challenged by both theoretical and empirical studies. A crucial question is whether and how internal noise limits perception in ASD, independently from other sources of perceptual inefficiency, such as the ability to filter out external noise. Here, we separately estimated internal noise and external noise filtering in ASD. In children and adolescents with and without ASD, we computationally modeled individuals’ visual orientation discrimination in the presence of varying levels of external noise. The results revealed increased internal noise and worse external noise filtering in individuals with ASD. For both factors, we also observed high inter-individual variability in ASD, with only the internal noise estimates significantly correlating with severity of ASD symptoms. We provide evidence for reduced perceptual efficiency in ASD that is due to both increased internal noise and worse external noise filtering, while highlighting internal noise as a possible contributing factor to variability in ASD symptoms.

## Introduction

Autism spectrum disorder (ASD) is a neurodevelopmental disorder characterized by deficits in social communication and the presence of restricted and repetitive behaviors^1^. The diagnostic criteria include atypical reactivity to sensory stimuli, which can manifest as either hyper- or hypo-sensitivity. For example, individuals with ASD frequently report extreme sensitivity to lights, sounds, and textures (hyper-sensitivity) or demonstrate decreased response to salient stimuli (hypo-sensitivity). In fact, the two states may co-occur, fluctuating across time within an individual^2^. This variability, both within and across individuals, presents substantial challenges to understanding the neural underpinnings of sensory processing in ASD.

In the typically developing (TD) population, perceptual variability can be explained as being derived from both the physical randomness in the external environment^3^ and internal variability in the neural system^4–7^. Environmental noise, often referred to as external noise, arises from inherent variations in sensory input. For instance, in vision, fluctuations in the number of photons emitted by a light source can fundamentally limit our ability to detect dim light^3^. However, even when such fluctuations in external input are effectively held constant, perception and associated behavioral responses still exhibit significant variability^8,9^. Such variability is in part caused by internal noise that broadly exists throughout the nervous system, which together with external noise, limits the quality and efficiency of perceptual processing^10^ (but see McDonnell & Ward^11^ for a notable exception).

In individuals with ASD, there is growing evidence that increased internal noise might play an important role in their behavioral symptoms^12–15^. Specifically, it has been hypothesized that elevated internal noise in ASD may produce atypically large fluctuations in neural responses, leading to unreliable and less predictable representations of the environment^16^. The idea is supported by fMRI and EEG studies that showed increased trial-by-trial variability in blood-oxygen-level dependent (BOLD) and electrical responses to sensory stimuli in ASD^17–19^. Dinstein *et al*.^19^ found that trial-by-trial variability in BOLD responses within visual, auditory, and somatosensory cortices in adults with ASD was increased compared to neurotypical controls, with no significant group differences in the average BOLD amplitude. A possible role of internal noise in contributing to behaviors associated with ASD is suggested by a recent study^20^, where neurotypical individuals with more autistic traits exhibited more variable responses to identical stimuli over multiple repetitions.

However, whether internal noise is actually elevated in individuals with ASD remains an open question. We do not yet have a good understanding of causes and computational principles that might underlie increased neural variability in ASD^21^, and several theoretical accounts suggest an opposite possibility that internal noise may be reduced in ASD^22,23^. Notably, a number of studies challenge the noisy brain hypothesis in ASD by demonstrating typical levels of variability in evoked EEG^24^ and MEG^25^ responses to sensory stimulation, as well as in psychophysically estimated internal noise^26,27^. At the core of this controversy lies the issue of whether a domain-general account of ASD, such as the ones involving elevated internal noise and neural variability, can truly explain the complex phenotypes in individual with ASD^24^. This has been further complicated by the fact that the estimates of neural variability have been based on responses to task-irrelevant stimuli^17–19,24,25^, making it difficult to estimate the degree to which internal noise limits perceptual performance in ASD.

Furthermore, the effects of internal noise have often not been adequately separated from neural processes associated with external noise^28^. As discussed above, neural variability arises from many different sources^10^, including the variability in the external environment. External noise can be effectively filtered out by relying on appropriate perceptual ‘templates’ that weigh task-relevant sensory information^9,29^. Using templates that are not suitable for a given task increases the influence of task-irrelevant information and leads to inaccurate and noisy representations of the environment^30^. In fact, recent studies showed that individuals with ASD are more impacted by the presence of task-irrelevant stimulus noise^27,31^, suggesting heightened sensitivity to external noise in ASD.

**Figure 1:**
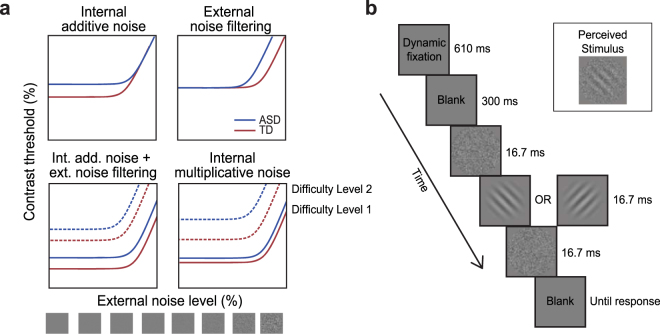
Predictions from PTM and experimental procedure. (**a**) Typical threshold-vs-noise (TvN) functions and predictions from the PTM. TvN functions have a characteristic shape where a flat segment is followed by a rising segment. At low external noise, internal noise dominates such that added external noise has little effect on perceptual performance. This produces the flat portion of the TvN curve. Once the external noise level begins to exceed that of internal noise, perceptual thresholds will be affected by external noise. Consequently, thresholds will increase because stronger signal is needed to overcome high levels of external noise. The PTM predicts distinct changes in TvN depending on the source of noise that is atypical in ASD (blue curves), relative to a TD baseline (red curves). Elevated internal additive noise will result in increased thresholds at the flat portion of the curve (top left). Worse external noise filtering, on the other hand, causes increased thresholds at the rising portion of the curve (top right). A combination of the two should yield increased thresholds across all levels of external noise levels (bottom left). A similar pattern is expected if internal multiplicative noise is elevated in ASD. However, the two can be distinguished by comparing the curves across at least two different difficulty levels. The ratio between thresholds at two difficulty levels will be different across groups in the case of elevated internal multiplicative noise in ASD (bottom right). (**b**) Stimuli, task, and timeline. Each trial began with a dynamic fixation point (see *Methods*). After a blank screen, the stimulus sequence started. Stimuli were oriented gratings temporally sandwiched by Gaussian pixel noise. This sequence yields merged perception of grating and noise (see insert). Participant’s task was to judge whether the grating was tilted left or right from vertical.

Given these gaps in knowledge, we aimed to quantitatively estimate different sources of noise that limit perceptual processing in individuals with ASD using psychophysical and computational methods. Specifically, we employed a well-established equivalent noise paradigm^32–36^ in the context of a simple visual orientation discrimination task. This paradigm allows us to computationally estimate the input noise ‘equivalent’ to the total amount of internal noise that constrains perceptual ability. In this framework, perceptual sensitivity is measured at varying levels of added external noise. The assumptions are that A) there is a constant amount of internal noise in the system, and that B) perceptual performance at a given external noise level is influenced by whichever noise (i.e., internal or external) dominates. An analogy would be one’s ability to hear at a noisy party. In the absence of any additional noise, the baseline party noise—corresponding to internal noise—will determine the limits of what one can hear. Added external noise (e.g., caused by air conditioning)—corresponding to external noise—will have negligible effects as long as its level is lower than the baseline party noise. However, if the added noise is strong (e.g., a malfunctioning air conditioner), it will start affecting the ability of partygoers to hear each other. Here, the negative effects of added noise can be mitigated by an appropriate perceptual template that excludes irrelevant noise in the sensory input—a process described as external noise filtering in the framework used here. Taken together, by measuring perceptual sensitivity across varying levels of external noise, we can separately characterize the influence of both internal and external noises on perception (Fig. 1a). Performance was modeled using the Perceptual Template Model (PTM)^36,37^ to quantitatively estimate different sources of noise that constrain perception.

The benefits of using equivalent noise paradigms to investigate internal noise in ASD have been previously highlighted^12,14^. In fact, two studies used a reduced version of this paradigm and found no significant evidence for elevated internal noise in ASD^26,27^. However, two important unanswered questions remain. First, given the extensive number of trials typically required for equivalent noise experiments, these previous studies estimated thresholds at only two noise levels (i.e., no-noise and high-noise). While the noise levels used are the two most diagnostic ones, such an approach is limited relative to the inferences that can be made by measuring a full threshold function over varying levels of external noise (Fig. 1a). Second, the previous studies used the equivalent noise approach in the context of direction or orientation integration across multiple stimuli. This is of relevance as visual integration relies on mechanisms distinct from those involved in processing of individual stimuli^38^. Moreover, internal noise for integration depends on the number of stimuli in the display^39^ and is shown to be even lower than that for processing a single item^40^. Thus, while these prior studies provide important insights on the visual integration abilities in ASD, it is not given that those results will generalize to other settings. Here, we aimed to estimate internal noise in the context of an ostensibly simpler stimulus and task. Specifically, we chose stimulus and task parameters to stay clear of known perceptual impairments in ASD. We used a coarse orientation discrimination task with larger stimulus size^41,42^ and low spatial frequency^43,44^. Our goal here was to minimize the influence of known atypicalities in ASD that are specific to processing of stimulus attributes per se. Additionally, to improve upon the methods previously used, we applied an advanced adaptive psychophysical technique to significantly increase the efficiency in data collection. The technique considerably reduced the time it takes to perform the experiment by 70%, which, together with hierarchical Bayesian model techniques, allowed us to behaviorally quantify the effects of noise on perception in ASD at both the group and individual levels.

## Results

### Perceptual Template Model and its predictions

We used an equivalent noise paradigm^32–36^ where perceptual thresholds are measured as a function of varying external noise levels added to the stimuli. This results in a characteristic threshold versus noise (TvN) function (Fig. 1a). To quantify the effects of noise on perception, data were fitted with the PTM^35,36^, a model where perceptual performance is limited by three different sources of inefficiency: external noise, internal additive noise, and internal multiplicative noise. Here, perceptual thresholds (*c*_*τ*_) are characterized by:

1 $${{\rm{c}}}_{\tau }=\frac{1}{\beta }{[\frac{(1+{N}_{mul}^{2}){N}_{ext}^{2\gamma }+{N}_{add}^{2}}{(1/d{\text{'}}^{2}-{N}_{mul}^{2})}]}^{\frac{1}{2\gamma }},$$

where the input (signal + external noise (*N*_*ext*_)) is filtered through a perceptual ‘template’ relevant to a given task (see *Supplementary Material* 1 for derivation). This results in signal enhancement by a gain factor *β*. The output of the filter is transformed through a nonlinear transducer function that amplifies the inputs to the *γ* th power, and various sources of internal noise—additive and multiplicative—are added. Internal additive noise (*N*_*add*_) remains constant at all signal levels (i.e., it is independent of signal). On the other hand, internal multiplicative noise (*N*_*mul*_) is proportional to the signal strength. Finally, a decision process determines the perceptual performance level (*d′* ). *N*_*ext*_ is manipulated by the experimenter along with the input signal.

Given the background detailed in the *Introduction*, we hypothesized that individuals with ASD have increased internal additive noise. This predicts worse perceptual thresholds at lower external noise levels (Fig. 1a, top left). Secondarily, we hypothesized worse external noise filtering in ASD^27,31^, which would impair performance at higher external noise levels (Fig. 1a, top right). A combined effect of higher internal additive noise and worse external noise filtering would be evident as increased thresholds in ASD at all external noise levels (Fig. 1a, bottom left). This expected pattern of results is similar to the effects of an increase in internal multiplicative noise. In the PTM, multiplicative noise is characterized similarly to contrast gain control mechanisms^36,37,45^. Increased multiplicative noise reduces the system’s response to stimulus contrast^29^, leading to worse performance across all external noise levels. However, because this effect is multiplicative, the expected result is a non-uniform shift in the TvN curve across different difficulty levels (Fig. 1a, bottom right; i.e., the ratio between the two difficulty levels will be different across groups)^29,37,45^. Therefore, to distinguish the effect of internal additive noise and external noise filtering from that of internal multiplicative noise, perceptual performance must be measured at multiple difficulty levels.

### Increased internal additive noise and worse external noise filtering in ASD

Twenty-one children and adolescents with ASD and 20 age- and IQ-matched TD controls completed an orientation discrimination task, judging whether a foveally presented grating (3° radius; Fig. 1b) was tilted to the left (−45°) or right (45°) from the vertical. External noise was manipulated by presenting Gaussian pixel noise with the same size as the grating immediately before and after stimulus presentation. Note that because of brief frames (16.7 ms) this sequence results in perceptual merging of grating and noise frames. This is typically done in PTM experiments to allow finer adjustments of target stimulus contrast^29,35,37^. Contrast thresholds at each of eight fixed external noise levels (0–21%) were measured. Stimulus presentation was controlled using an advanced adaptive psychophysical technique^46^. As detailed in *Methods*, this allowed us to accurately estimate relevant model parameters in just 480 trials per participant.

**Figure 2:**
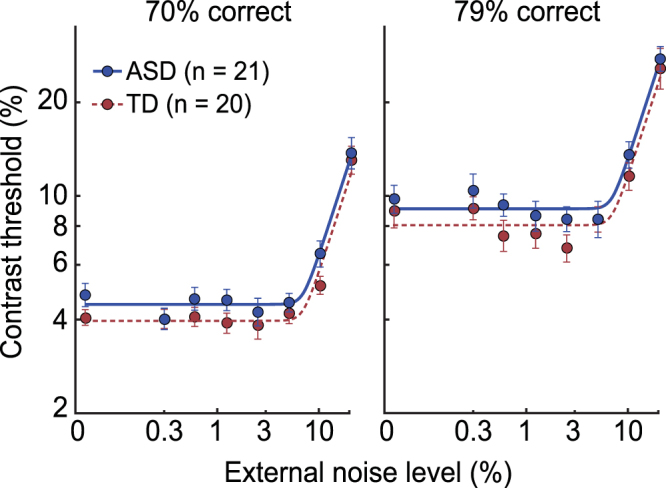
Results from the conventional PTM analysis. The two panels show thresholds (filled dots) at each external noise level for two difficulty levels. Overall, individuals with ASD (blue) performed worse than those with TD (red) across external noise levels. Error bars are ±SEM. Curves were obtained by fitting the conventional PTM (Equation (3) in *Methods*), which revealed a 70% increase in internal additive noise and 13% worse external noise filtering in ASD (p < 0.001; compared to the model that assumed no group differences).

We first analyzed the data using the conventional PTM (*Methods*) which allows us to estimate the *relative* difference in noise-limiting factors between groups^36^. TvN curves estimated by the conventional PTM analysis revealed the characteristic non-linear patterns for both ASD and TD (Fig. 2). On average, individuals with ASD had a tendency to perform worse than TD across external noise levels. This pattern of results was best explained by a combined effect of a 70% increase in internal additive noise in ASD paired with 13% worse external noise filtering (r^2^ = 98.2%). This model was significantly different from a model that assumed no group differences in model parameters (F_2, 26_ = 9.41, p < 0.001), but was not statistically different from a full model that assumed all noise sources were different between the groups (F_1, 25_ = 0.139, p = 0.71). These results support the hypothesis that individuals with ASD have both elevated internal additive noise and worse ability to filter out external noise in the stimuli. We found no evidence for group differences in internal multiplicative noise.

Group differences in independently estimated contrast thresholds at each external noise level, however, did not reach significance (main effect of group; F_1, 39_ = 2.05, p = 0.16). This was expected. Given that our empirical approach was optimized for estimating relevant model parameters with as few individual trials as possible, independent threshold estimates were noisy (a more sensitive analysis of group differences in thresholds is presented in the next sub-section). We also calculated the Jeffreys, Zellener, and Siow (JZS) Bayes factor to quantify the evidence for the null hypothesis versus the alternative, using the prior on effect size following a Cauchy distribution with a scale factor of 0.707 as recommended by Rouder *et al*.^47^. By convention, Bayes factors ranging between 0.33 and 3 indicate data insensitivity while those below 0.33 or above 3 suggests that data supports the null or the alternative hypothesis, respectively^47,48^. The calculated Bayes factor was 0.7, suggesting that comparison of individual thresholds was not sensitive and that the alternative hypothesis cannot be fully excluded. Turning to the multiplicative noise, both groups had similar ratios of thresholds at two difficulty levels (ASD: mean = 0.53, SD = 0.09; TD: mean = 0.54, SD = 0.11; t_39_ = 0.34, p = 0.74). The JZS Bayes factor for the group difference in threshold ratio was 0.32. These findings are consistent with a lack of group differences in multiplicative noise.

### Individual differences in internal noise and external noise filtering

**Figure 3:**
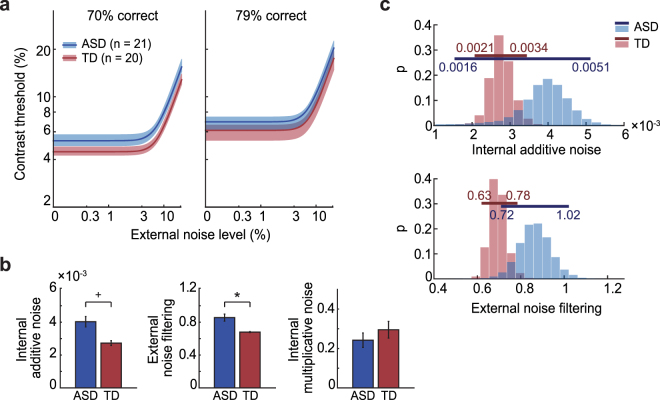
Results from hierarchical Bayesian analysis. (**a**) TvN functions estimated from the hierarchical Bayesian analysis show worse performance in ASD (blue) compared to TD (red), consistent with the conventional PTM analysis. Shaded regions are the 68% credible intervals obtained from the posterior distributions. (**b**) The average individual parameter estimates from the hierarchical Bayesian model. The estimated population means show 48% elevated internal additive noise, and 25% greater effect of external noise (i.e., worse external noise filtering) in ASD (+marginal significance using a 90% criterion on difference in population posterior samples; *significance using a 95% criterion). No significant group difference was found for internal multiplicative noise. Note that the absolute values of different types of noise cannot be meaningfully compared. Error bars are ±SEM. (**c**) Population posterior distributions estimated from the hierarchical Bayesian model for internal additive noise (top) and external noise filtering (bottom). Horizontal lines indicate 95% credible intervals. In both cases, the population posterior distributions for ASD (blue) are broader than TD (red), indicating greater inter-individual variability in ASD. In the case of internal additive noise, the 95% credible interval for ASD fully includes and extends beyond the credible interval for TD.

TvN curves and parameter estimates from the hierarchical Bayesian model exhibited a similar pattern to those estimated from conventional PTM (Fig. 3a,b). As this analysis removes some of the noise in threshold estimates by assuming that the variability between subjects in model parameters follows a population-level distribution^49,50^, we re-examined group differences in thresholds. Notably, individuals with ASD performed worse overall across external noise levels (F_1,39_ = 4.39, p = 0.04). There was no significant interaction between group and external noise level (F_7, 273_ = 0.42, p = 0.89), or between group and difficulty level (F_1,39_ = 0.5, p = 0.48). The estimated internal additive noise was 48% higher in ASD (population mean = 0.004) compared to TD (population mean = 0.0027). This group difference, however, was marginal (95% credible interval on difference = −0.0012, 0.0025; 91.3% of population posterior samples greater than zero). Here, the large increase in internal noise estimates was paired with greater individual variability in the ASD group (Fig. 3c, top). The 95% credible interval in the population posterior distribution for internal additive noise in ASD (0.0016, 0.0051) was 2.7 times broader than that in TD (0.0021, 0.0034). We will return to this individual variability when considering links with ASD symptoms. Turning to external noise filtering, we found that ASD was characterized by 25% worse external noise filtering abilities (population mean = 0.86) compared to TD (population mean = 0.69; 95% credible interval on difference = 0.003, 0.34; 97.6% of population posterior samples greater than zero). As with internal additive noise, we observed large individual variability (Fig. 3c, bottom), with the 95% credible interval in the population posterior distribution for external noise filtering in ASD (0.72, 1.02) being 2.0 times broader than in TD (0.63, 0.78). Finally, the two groups were not different in internal multiplicative noise (population mean for ASD = 0.11; for TD = 0.26; 95% credible intervals on difference = −0.48, 0.41; 42.8% of population posterior samples greater than zero).

### Internal additive noise is correlated with ASD symptoms

**Figure 4:**
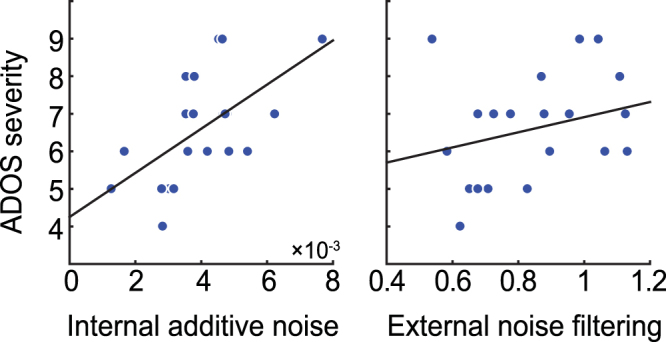
Relationship between the model parameters and ADOS calibrated severity score within ASD. Correlation analyses indicated that symptom severity was related to internal additive noise (left panel; r_19_ = 0.59, p = 0.005) and not to external noise filtering (right panel; r_19_ = 0.28, p = 0.22; see main text for results from multiple regression analysis which include both noise estimates in the model).

There was no significant correlation between internal additive noise and external noise filtering within ASD (r_19_ = 0.19, p = 0.42), suggesting, at least in part, independent contributions of these two sources of noise to perceptual inefficiencies in ASD. To test for links with behavioral ASD symptoms, we tested whether our noise estimates can predict individuals’ calibrated severity score derived from the Autism Diagnostic Observation Schedule (ADOS). Severity scores ranged from 4–9 on this dimensional measure of ASD severity, where scores of 4 and above are indicative of ASD (see Fig. 4 for simple correlations between symptom severity and each of the noise parameters). We then conducted a multiple linear regression analysis in the ASD group to understand the unique contributions of each noise type (estimated model parameters of internal additive noise and external noise filtering) on ADOS severity. Together, internal additive noise and external noise filtering accounted for 47% of the variance in ADOS severity score (R^2^ = 0.38, F_2,18_ = 5.56, p = 0.01). The results indicated that internal additive noise uniquely and significantly predicted the ADOS severity score in this combined model (β = 555.88, t_18_ = 2.98, p = 0.008). Specifically, higher internal additive noise was associated with increased severity of assessed ASD symptoms. On the other hand, external noise filtering did not significantly predict the ADOS severity score (β = 1.25, t_18_ = 0.91, p = 0.37). The results did not change when IQ was included as a predictor in the analysis. Finally, given the correlation between internal additive noise and ASD symptom severity, we repeated our analysis on the independently estimated Weibull thresholds by comparing the TD participants with those individuals with ASD who had more severe symptoms (median split using ADOS severity score, n = 11). This revealed a main effect of group (F_1,29_ = 4.78, p = 0.04) where this more severe ASD group performed significantly worse than the TD group. Taken together, these results suggest that the psychophysically estimated internal additive noise might be linked to the severity of core behavioral symptoms of ASD and support our hypothesis that, for basic visual processing, individuals with ASD have elevated internal noise.

## Discussion

The present study utilized psychophysical and computational methods to elucidate the sources of noise that influence perceptual performance in individuals with ASD. We found evidence for elevated internal noise and poor external noise filtering in ASD. Moreover, the estimated internal noise was correlated with ASD symptom severity, suggesting that individual variability in internal noise may be related to ASD symptomatology. Although the observed perceptual effect was relatively small (6.91% average increase in thresholds in ASD), evidence for higher internal noise in ASD is consistently observed across our model analyses. These findings support current theories that argue for an important role of increased internal noise in atypical sensory abilities in ASD, and suggest external noise filtering as another possible source of perceptual variability in ASD.

Our findings that individuals with ASD have elevated internal noise at the group level is consistent with previous studies that showed increased trial-by-trial variability in BOLD and EEG responses to sensory stimuli in ASD^17–19^ (but see Butler *et al*.^24^ and Coskun *et al*.^25^). While trial-by-trial variability in neural responses in the sensory areas is likely driven by a number of factors, noise levels in the nervous system are a key determinant^10^. Internal noise is also an important factor in perceptual variability^5,8^. Individuals with ASD report more varying perceptual judgments to repeated presentations of a sensory stimulus^51^. Such perceptual and neural fluctuations may have a substantial effect on the reliability of sensory representations, which in turn may cause greater difficulties for individuals with ASD when interacting with the surrounding environment. We also found significantly worsened external noise filtering in ASD. This may reflect abnormalities in the ability to extract task-relevant structures from visual inputs. In the typical population, there exist individual differences in the use of noise-excluding ‘templates’, which are believed to reflect variations in the ability to extract task-relevant information^52^ and to integrate the outputs of lower-level sensory channels into decision^45^—an ability that can be improved through perceptual learning^29,53^.

The present study extends the existing literature on internal noise in ASD in several important ways. Unlike recent studies that were based on brain responses to task-irrelevant stimuli^17–19,24^, our estimates of internal noise were functionally relevant to the task at hand (i.e., specifically associated with the limitations in visual perception in ASD). This is notable because of known atypicalities in task-dependent changes in neural processing in ASD^54^. However, this also raises a question of generalizability of our findings to other task settings. By definition, estimates of internal noise index the total amount of noise that is equivalent to the observer’s intrinsic noise. This intrinsic noise could be derived from sources specific to processing stimulus attributes required by a given task^55^. For example, internal noise estimates can be substantially elevated within an individual if the task/stimulus involves a known impairment^56^. On the other hand, a portion of estimated internal noise should also reflect the baseline noise that can have limiting effects on a wide range of processes within a system. In this study, we utilized a coarse orientation discrimination task that relies on the measurement of contrast thresholds with low spatial frequency stimuli whose processing is largely intact in ASD^43,44^. This makes it less likely that our internal noise estimates are elevated due to selective impairments associated with our specific task and stimuli, and suggests that perception may be limited by the same noise in other settings.

Our study presents discrepant findings from previous studies that used similar equivalent noise paradigms and did not find atypical levels of internal noise in ASD^26,27^. There are several possible reasons for this difference. First, as discussed in the *Introduction*, these studies used an impoverished version of the equivalent noise paradigm, relying on only two noise levels. While the selected noise levels were the most diagnostic ones, this approach precludes the measurement of full TvN curves and, consequently, application of arguably more sensitive model analyses used in the present study. Second, the nature of the task, namely an explicit need for stimulus integration in prior studies, may play a role in the observed discrepancy. While the ecological importance of visual integration processes motivates its study in context of ASD, visual integration also involves distinct mechanisms which are known to have differential impacts on the estimated internal noise^40^. In addition, a third factor that should be considered is the variability within individuals with ASD themselves^21^, which is frequently observed in ASD^42,57–62^. In fact, enhanced orientation sensitivity in ASD has been reported^60^, which would predict lower internal noise in ASD based on our assumptions in the model. This is in contrast with the previous account of internal noise in ASD (e.g., stochastic resonance) which argues that elevated internal noise, as a global trait of ASD, can explain both enhanced and diminished perceptual sensitivity in this population^12^. Instead, our findings reveal large inter-individual variability in both internal additive noise and external noise filtering among ASD. While such variability in ASD results emphasizes the need for replication of our findings, this also raises an important possibility that subgroups may exist within the ASD population that are characterized by different levels of internal noise. Our observation that internal noise estimates were positively related to severity of ASD symptoms, as well as a previous report of the link between internal noise and autistic traits^20^, further supports this idea.

An alternative explanation of our findings may involve deficits in attentional deployment in ASD. In TD, spatial attention can reduce the effects of internal additive noise, without affecting external noise filtering^37^. While only partially consistent with our results, this still raises a possibility that increased internal additive noise in ASD may result from impairments in spatial attention. However, previous reaction time studies report mixed findings on spatial attention deficits in ASD (for a review: Ames and Fletcher-Watson^63^). Moreover, the studies that directly investigated the effects of spatial attention on visual performance in ASD found either no significant deficits^64,65^ or, in one case, a sharper window for spatial attention in ASD^66^. Additionally, if our results were caused by group differences in global attention or task motivation, we would expect similar effects on internal noise and external noise filtering estimates—an expectation inconsistent with a lack of significant correlation between the two noise factors. Therefore, although we cannot completely rule out effects of attention, it is unlikely that attentional differences would cause the specific pattern of results observed in this study.

Our findings are relevant to the growing literature on Bayesian perceptual priors^2^ and predictive coding in ASD^67,68^. Bayesian priors are helpful in attenuating the effects of noise^69^, and play an important role in constraining sensory noise throughout development^70^. In this context, atypically strong effects of noise in ASD may, in part, reflect diminished reliance on priors^31,71^. Supporting this idea, several studies report reduced adaptation in ASD across multiple domains^72–74^, suggesting another example of perceptual inefficiency in this population. Such an account is also consistent with the weaker divisive normalization hypothesis in ASD, which can lead to reduced influence of contextual information^75^. On the other hand, unreliability in sensory representations due to noisier processing may also prevent individuals from forming reliable priors. In a predictive coding framework, noisier perceptual processing can be explained by individuals’ tendencies to more heavily weigh prediction errors caused by irrelevant information^68,76^. Indeed, a recent empirical finding suggests that individuals with ASD have an increased tendency to attribute aberrant events to changes in the environment^77^. This can increase unpredictability in perception, and might be further related to the desire for sameness frequently observed in ASD^68,78^. Thus, future research should consider how the influence of noise in each individual relates to the use of top-down information such as prior and prediction, as well as their possible interactions across development.

In conclusion, we report evidence for elevated internal noise and poor external noise filtering in ASD. Our results further reveal large inter-individual variability in estimated levels of internal noise and external noise filtering, the former of which is related to severity of ASD symptoms. Evidently, visual processing in ASD may be less efficient, limited by multiple sources of noise. These results are pertinent to existing theories on atypical sensory processing in ASD, providing important insights about the constraints of reliable perceptual representations that could have substantial effects on individuals’ everyday lives. Future studies should investigate how our findings relate to neurophysiological measures of noise and generalize to other sensory modalities.

## Methods

### Participants

Participants were 21 children and adolescents (ages 9–17 years) with ASD (19 male) and 20 TD controls (18 male). This sample size was determined to be the larger of (a) previous studies that investigated perceptual efficiencies across groups using the PTM^56,79^ and (b) the typical sample size in studies that examined various aspects of sensory processing in ASD (~20 participants in a group)^80–84^. We assessed IQ using abbreviated versions of the Wechsler Intelligence Scale for Children, 4^th^ Edition^85^ or the Wechsler Adult Intelligence Scale, 4^th^ Edition^86^. Groups were matched on both age (ASD: mean age = 13.49, SD = 2.08; TD: mean age = 13.47, SD = 2.31; t_39_ = 0.02, p = 0.98) and full scale IQ (ASD: mean FSIQ = 110.14, SD = 14.14; TD: mean FSIQ = 113.70, SD = 15.10; t_39_ = 0.78, p* = *0.44). ASD diagnosis was confirmed or ruled out with a combination of the ADOS^87^, and either the Autism Diagnostic Interview–Revised (ADI-R)^88^ with parents of participants with ASD or the Social Communication Questionnaire (SCQ)^89^ with parents of TD participants. The ADOS and ADI-R were administered by a research-reliable examiner and a licensed clinical psychologist made final diagnostic decisions. From the ADOS, we derived the calibrated severity score^90,91^ (range: 4–9) to use in our correlation analyses. Thirteen of the participants with ASD were taking medications at the time of the study, which included stimulants and other psychoactive medications (e.g., guanfacine, fluoxetine). There were no effects of medication on any of our experimental measures within ASD (all p’s > 0.4), and no effects of medication on correlations between our measures and symptom severity. However, given the limited power we have to investigate medicated versus un-medicated participants, we cannot completely rule out effects of medication. All parents gave written informed consent, and participants gave assent. All were paid for participation. Procedures were conducted according to a protocol approved by the University’s Research Subjects Review Board.

### Stimuli, task, and experimental procedure

Stimuli were created using MATLAB and Psychophysics Toolbox^92^. They were shown on a linearized monitor (24-inch Sony GDM-FW900 CRT; 1024 × 640 resolution; 120 Hz). Viewing distance was 77 cm with each pixel subtending 0.036°, and a chin rest was used. An experimenter was present in the room throughout to encourage on-task behavior.

Participants performed a coarse orientation discrimination task where they judged the tilt of the stimulus (±45° from the vertical; Fig. 1b). The stimulus was a sinewave grating (1 cycle/°) presented at the center of the display in a two-dimensional raised cosine envelope (radius = 3°). The stimulus was temporally sandwiched by two independent external noise samples (Fig. 1b). The grey-level pixel values for external noise (each subtending 0.14° × 0.14°) were sampled from a Gaussian distribution with a mean of 0 and SD that varied over trials, such that its root mean square (RMS) contrast defined the external noise level (0, 0.3, 0.61, 1.24, 2.51, 5.1, 10.3, or 21%). A trial began with a dynamic fixation sequence presented at the center of the screen^62^. Participants indicated their response by pressing the left or right arrow key. Auditory feedback followed correct responses.

### Psychophysical procedure

Stimulus contrast was adjusted on every trial to estimate the signal strength required to reliably perceive stimulus orientation for a given external noise level. To do this, we used an advanced adaptive psychophysical method, Functional Adaptive Sequential Testing (FAST toolbox; Vul *et al*., 2010), to efficiently sample stimulus contrast around participants’ contrast thresholds. FAST optimally and adaptively selected a stimulus contrast that is most informative for estimating the model parameters. We implemented two interleaved FAST structures that independently estimated psychophysical functions at two different difficulty levels (*d*′) using Equation (1). For each of the two FAST structures, *d*′ was set at 1.089 and 1.634, corresponding to 70.71% and 79.37% difficulty levels, respectively. Unlike conventional approaches that first aim to estimate thresholds at each external noise level, FAST substantially increases data collection efficiency by directly constraining the shape of the full TvN function using all data points^46^. Compared to conventional methods that require ~1500 trials^29^, this technique allowed us to test our hypotheses in a more efficient manner, requiring only 480 trials total for each participant (~25 minutes). We settled on this design after extensive piloting, first in adults and then in children. For this, we conducted longer experiments and estimated the number of trials at which estimates of the PTM parameters asymptoted. Similar techniques have been shown to be effective for testing visual performance in TD children^93^.

Note that the data analyses were performed independent from the FAST procedure. This was to minimize possible effects of erroneous responses in ASD, which could result in biased threshold estimations if estimated directly using these types of adaptive techniques^12,14^. Specifically, we analyzed the data in two complementing ways as described below. First, we estimated model parameters by fitting the conventional PTM, with an aim to test for *group* differences in internal additive noise, internal multiplicative noise, and external noise filtering^29,37^. Second, we directly estimated model parameters from single trial data using hierarchical Bayesian model fitting. This method estimated model parameters for each *individual* participant, providing a better index of individual differences^94^. This further allowed us to test for links between behavioral ASD symptoms and noise-related perceptual limitations.

### Conventional PTM analysis

To analyze the data using a conventional PTM fitting method, we first estimated psychophysical thresholds for each participant at each of the external noise levels. We pooled the data from the two FAST structures to estimate thresholds, resulting in 60 trials for each external noise level. This yielded independent, albeit not very precise, threshold estimates at each noise level. Thresholds were estimated by fitting a Weibull function at each external noise level independently:

2 $$P(c)=1-(1-0.5)\times {2}^{-{(\frac{\mathrm{log}(c)}{\alpha })}^{\eta }},$$

where *P* denotes percent correct, *c* is stimulus contrast, *α* is threshold at 75% performance level and *η* is the slope. To estimate the two free parameters of the Weibull function (*η* and *α*), we used a Bayesian model fitting method that implements a Markov Chain Monte Carlo (MCMC) technique. Specifically, we sampled the posterior distributions of the parameters using JAGS software (http://mcmc-jags.sourceforge.net/). We assumed a broad uniform prior on each parameter with a range that includes all practically possible values. Maximum a posteriori (the mode of the posterior) were used as the best estimates of the model parameters. We discarded the first 15,000 samples as a burn-in period, and thinned the samples to reduce correlations by only selecting every 200 samples. A total of 10 chains were run in parallel, resulting in 1,000 posterior samples from each chain. Thresholds at 70.71% and 79.37% were then computed from the estimated Weibull functions.

The group averages of the independently estimated thresholds were then used to fit the conventional PTM. To characterize the group difference, the PTM introduces three coefficient indices (*A*_*m*_(*group*), *A*_*a*_(*group*),*A*_*f*_(*group*)) to Equation (1), each to be multiplied by *N*_*mul*_, *N*_*add*_, and *N*_*ext*_:

3 $${c}_{\tau }=\frac{1}{\beta }{[\frac{(1+{({A}_{m}(group){N}_{mul})}^{2}){({A}_{e}(group){N}_{ext})}^{2\gamma }+{({A}_{a}(group){N}_{add})}^{2}}{(1/{d}^{\text{'}2}-{({A}_{m}(group){N}_{mul})}^{2})}]}^{\frac{1}{2\gamma }}.$$

The coefficient indices for TD are fixed to be *A*_*m*_(*TD*) = *A*_*a*_(*TD*) = *A*_*e*_(*TD*) = 1. To estimate group differences in how the three types of noise influenced performance, *A*_*m*_(*ASD*), *A*_*a*_(*ASD*), and *A*_*e*_(*ASD*) are varied. That is, these coefficients for ASD describe the *relative* differences in the effects of noise between the groups. Specifically, if the estimated *A*_*m*_(*ASD*), *A*_*a*_(*ASD*) and *A*_*e*_(*ASD*) are greater than 1, it suggests that the amount of corresponding types of noise in ASD is higher than TD. The three coefficient indices for ASD could potentially vary or be fixed at 1, resulting in eight candidate models to compare. For example, the null model, assuming no group differences, had only four free parameters (*N*_*mul*_, *N*_*add*_, *β* and *γ*), whereas the full model, assuming group differences in all three types of noise, had seven free parameters (*A*_*m*_(*ASD*) and *A*_*a*_(*ASD*), and *A*_*e*_(*ASD*), in addition to the four in the null model). The remaining six candidate models had different combinations of the three types of noise, resulting in five or six free parameters. Each of the candidate models was fitted to the average group thresholds estimated from the above-described method using a least-squares procedure.

For model comparison, goodness-of-fit for each candidate model was evaluated using *r* ^2^:

4 $${r}^{2}=1-\frac{\sum {[\mathrm{log}({c}_{\tau }^{theory})-\mathrm{log}({c}_{\tau })]}^{2}}{{\sum [\mathrm{log}({c}_{\tau })-mean(\mathrm{log}({c}_{\tau }))]}^{2}},$$

where Σ and *mean* were computed over all difficulty levels, external noise levels, and groups. The best-fitting model was identified using the *F*-test for nested models:

5 $$F(d{f}_{1},d{f}_{2})=\frac{({r}_{full}^{2}-{r}_{reduced}^{2})/d{f}_{1}}{(1-{r}_{full}^{2})/d{f}_{2}},$$

where *df*_*1*_ = *k*_*full*_ − *k*_*reduced*_ and *df*_*2*_ = *N*_*data*_ − *k*_*full*_. *k*_*full*_ is the number of free parameters in the full model, and *k*_reduced_ is the number of free parameters in reduced models. *N*_*data*_ is the number of predicted data points. The model with the fewest number of free parameters that was not significantly different from the full model was determined to be the best-fitting model. A significance level of α < 0.05 was used.

### Hierarchical Bayesian model analysis

To test for links between ASD symptoms and individual variability in the noise estimates, we fitted the PTM for each participant using a hierarchical Bayesian modeling technique. By assuming that each participant is drawn from a population distribution (ASD or TD), this method increases statistical power^49,50^. Importantly, for our purposes, it allows estimation of model parameters for each participant within a population, providing a better understanding of individual differences^94^. Furthermore, using this approach, we can estimate model parameters directly from single trial data points (instead of relying on the independently estimated thresholds at each external noise level).

We used a variation of PTM that minimized the number of free parameters and allowed us to estimate the effects of all three types of noise for each participant. Specifically, we assumed that an individual’s response (*response*_*ijk*_), whether correct or incorrect, for a given trial is drawn from a Bernoulli distribution:

$$respons{e}_{ijk}\sim Bernoulli({\theta }_{ijk}),$$

where *i* is difficulty level, *j* is external noise level, and *k* is trial number. The probability of making a correct response (*θ*_*ijk*_) is given by:

6 $${\theta }_{ijk}=1-(1-0.5){e}^{{({m}_{i}\mathrm{log}({x}_{ijk})/\mathrm{log}({c}_{\tau ij}))}^{\eta }},$$

where $x$ is stimulus contrast, *η* is slope, and

7 $${m}_{i}=-\mathrm{log}\,{(\frac{1-{q}_{i}}{1-0.5})}^{\frac{1}{\eta }}.$$

The parameter *q* is the difficulty level and thus is either 0.7071 or 0.7937. Following the PTM, the perceptual threshold, ${c}_{{\tau }_{ij}}$, is defined by:

8 $${c}_{{\tau }_{ij}}=\frac{1}{\beta }{[\frac{(1+{N}_{mul}^{2}){({w}_{ext}{N}_{ex{t}_{ij}})}^{2\gamma }+{N}_{add}^{2}}{(1/d{\text{'}}_{i}^{2}-{N}_{mul}^{2})}]}^{\frac{1}{2\gamma }}.$$

Equation (8) is identical to Equation (1) except that a coefficient for external noise (*w*_*ext*_) is added to characterize the influence of external noise on the individual’s perception (i.e., extent of external noise filtering; see *Supplementary Material* 1 for derivations). For each participant, we estimated three parameters *N*_*mul*_, *N*_*add*_, and *w*_*ext*_ that were the most relevant for our hypotheses, in addition to the slope of the psychometric function (*η*). To do so, we simplified the model by assuming *β* = 1.25 for all participants, and further set *γ* = 2. We note that setting these two parameters to different values did not change the results (*Supplementary Material 1* for details). The fixed values we used are within a reasonable range reported in previous studies^29,36,37,45,56^. This also simplified the model by eliminating strong correlations among model parameters (see *Supplementary Material* 2, *Supplementary* Figs. 1 and 2 for additional details). In other words, we ensured that all model parameters are identifiable and sufficiently constrained based on the available data.

Similar to the Weibull fitting procedure, an MCMC technique was used to sample from the posterior distributions and estimate the free parameters. Here, we assumed hierarchical priors on each of the model parameters. That is, each model parameter that characterizes an individual participant’s data was assumed to be drawn from an independent Gaussian population distribution with a mean and SD that characterized each group. The priors for these population means and SDs were set to broad uniform distributions. Maximum a posteriori were used as the best parameter estimates for both individual and population parameters (using the mean did not change the results). To test the group difference, 95% credible intervals on the population posterior samples were used^95^. When the credible interval for the group difference (ASD-TD) does not include zero, the null hypothesis that there is no group difference is rejected. We used 15,000 iterations for burn-in, and only selected every 200 samples for thinning. Ten chains were run in parallel, each of which sampled 2,000 posterior samples (see *Supplementary Material* 3 and *Supplementary* Fig. 3 for sample convergence and autocorrelation). To test the link between the noise-related perceptual limitations and ASD symptoms, we used the estimated individual model parameters to correlate with the calibrated severity score derived from the ADOS. A significance level of α < 0.05 was used for correlation analyses.

### Data availability

The datasets generated during and/or analyzed during the current study are available from the corresponding author on reasonable request.

## Electronic supplementary material

Supplementary Material
