# Supplementary material for: High internal noise and poor external noise filtering characterize perception in autism spectrum disorder
Source: Sci Rep. 2017 Dec 14;7:17584. doi: 10.1038/s41598-017-17676-5 (PMC5730555; doi:10.1038/s41598-017-17676-5)
Supplement: Supplementary file 1 — Supplementary Material [file 41598_2017_17676_MOESM1_ESM.pdf]

Supplementary Material

High internal noise and poor external noise filtering characterize perception in autism spectrum disorder

Park WJ, Schauder KB, Zhang R, Bennetto L, Tadin D

Table of Contents

1. Derivation and assumptions of Equation [8]

2. Correlation among parameters in the hierarchical Bayesian model

Figure S1. A bivariate correlation matrix plot for the model parameters with strong correlations in the hierarchical Bayesian PTM when all parameters are free

Figure S2. A bivariate correlation matrix plot for the model parameters in the hierarchical Bayesian PTM with fixed signal gain and nonlinearity

3. Convergence and autocorrelation in posterior samples

Figure S3. Trace plots and autocorrelation functions for the model parameter samples in the hierarchical Bayesian PTM obtained via the MCMC procedure

References

1. Derivation and assumptions of Equation [8]

In PTM, it is assumed that the input signal and external noise are processed through a perceptual template and a rectified nonlinear transducer function, the output of which is then added with various sources of internal noise to make perceptual decisions. A perceptual template is a linear spatio-temporal integrator. For a fixed signal $S_0(x, y, t)$ and template $T(x, y, t)$ defined over space and time, the template-matched signal (T_{s0}) yields

$$T_{s0} = \iiint T(x, y, t) S_0(x, y, t) dxdydt \ ,$$

where $\iiint S_0^2(x, y, t) dxdydt = 1$. Similarly, external noise, $G(x, y, t)$, where the value at each (x, y, t) point is drawn from a Gaussian distribution with a mean of 0 and standard deviation of σ_{ext}, is also processed through the same template, yielding

$$T_G = \iiint T(x, y, t) G(x, y, t) dxdydt \ .$$

Both the stimulus and external noise are modulated by contrast, such that a contrast-modulated stimulus (S) and external noise ($N(x, y, t)$) becomes $S = cS_0(x, y, t)$ and $N(x, y, t) = N_{ext} G(x, y, t)$, where c and N_{ext} respectively indicate the contrast of stimulus and external noise. Thus, the template-matched signal (T_s) and external noise (T_n) yields

$$T_s = c \iiint T(x, y, t) S_0(x, y, t) dxdydt = cT_{s_0}$$

$$T_n = N_{ext} \iiint T(x, y, t) G(x, y, t) dxdydt = N_{ext} T_G \ .$$

T_{s_0} is a constant, characterizing the intrinsic template-matched signal. T_G is a random variable drawn from a Gaussian distribution with a mean of 0 and a fixed standard deviation of σ_{T_G} such that

$$T_n = N_{ext} \sigma_{T_G} G(0, 1) \ ,$$

where $G(0, 1)$ is a sample from a standard normal distribution. Mathematically, because T_{s_0} and T_G can be known only up to a constant, without losing generality, the PTM conventionally simplifies the model by assuming $\sigma_{T_G} = 1$ for the baseline (i.e., intervention neutral) state. It is equivalent to assuming that the integral of the total gain for the perceptual template at baseline is normalized to 1 such that $T_s = \beta c$, and $T_n = N_{ext} G(0, 1)$, where $\beta = T_{s_0} / \sigma_{T_G}$. Such assumption eliminates the necessity of keeping $N_{ext} \sigma_{T_G}$ throughout the equations[1]. This has been also useful

in investigating, for example, the effects of attention and perceptual learning in typical populations[2,3]. In our conventional PTM analysis, we follow this assumption and characterize the *group* difference in external noise filtering between ASD and TD. To do this, the PTM introduces a coefficient (A_f) as in Equation [3]. By setting $A_f(TD) = 1$, the estimated $A_f(ASD)$ describes the *relative* difference in the influence of external noise in ASD compared to TD. This is analogous to characterizing the difference between attention (or post-learning) and no attention (or pre-learning).

In the hierarchical Bayesian analysis, however, we relax the above assumption, as our primary purpose is to characterize the *individual* differences in external noise filtering in ASD. That is, we separately characterize the gains in signal and external noise such that $\beta = T_{s_0}$, and $w_{ext} = \sigma_{T_G}$ (Equation [8] in the main text). This modification allows us to estimate the external noise filtering in each individual. The remaining derivation of the model (e.g., nonlinear transducer function $\|\cdot\|^{\gamma}$, characterization of internal additive N_a and multiplicative noise N_m, decision criteria, and threshold function) is identical to previous studies, and can be found elsewhere[1,3,4]. We further simplify the model by fixing β and γ, because of high positive correlations between β and N_a, and between β and w_{ext}, as well as a negative correlation between γ and N_a (see *Supplementary Material* 2 for more details). Setting β to different values (e.g., 1.98; our estimate from conventional PTM) only scaled other model parameters and did not change the results. The same results were obtained even when $\beta = 1$ (i.e., eliminating the parameter). Note that, while β indicates signal gain, any differences in contrast gain in ASD should also affect their responses to external noise, and thus will be reflected in N_{mul}. Also, setting γ to different values (e.g., 4.01; estimate from conventional PTM) did not cause qualitative changes in the results. We note that the fixed values we used are within a reported range[1–3,5,6].

2. Correlation among parameters in the hierarchical Bayesian model

We simplified the hierarchical Bayesian model by setting signal gain (β), and nonlinearity (γ) to a fixed value (see *Supplementary Material* 1). Such constraints were necessary because of strong correlations among the model parameters when the two parameters were free. Since the PTM is a complex model, it is important to check and ensure that the

parameters are identifiable and sufficiently constrained based on the available data. Figure S1 shows a bivariate correlation matrix plot for the internal additive noise (N_a), external noise filtering (w_{ext}), β, and γ for a representative TD participant. Each data point is a sample from the posterior distribution obtained via the Monte Carlo Markov Chain (MCMC) sampling procedure. There are strong correlations particularly between w_{ext} and β, N_a and β and N_a and γ. These correlations across parameters can make it difficult to constrain model parameters, and indicate that the data can be explained with a fewer number of parameters. Figure S2 depicts a bivariate correlation matrix for N_a, N_m, w_{ext}, and slope of the psychometric function (η) with a fixed β and γ (as reported in the main text). Data points are from posterior samples obtained for a participant in each group (blue: ASD; red: TD). None of the parameters are highly correlated with each other, and the distribution of the samples show that the two participants differ in N_a and w_{ext} (best illustrated by the second panel in the top row). Note, when we estimated model parameters for individual participants (e.g., Figure 4 in main text), the mode of the distributions shown in Figure S3 is taken as the parameter estimate (similar data were obtained for mean estimates).

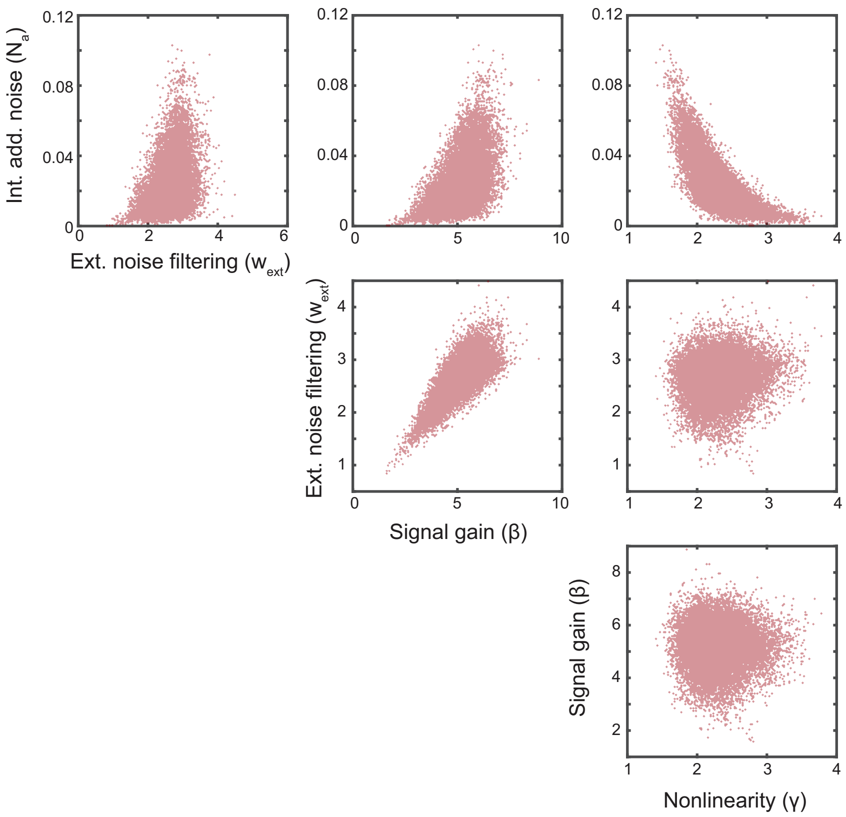

Figure S1. A bivariate correlation matrix plot for the model parameters with strong correlations in the hierarchical Bayesian PTM when all parameters are free. Data points represent samples from a representative participant with TD.

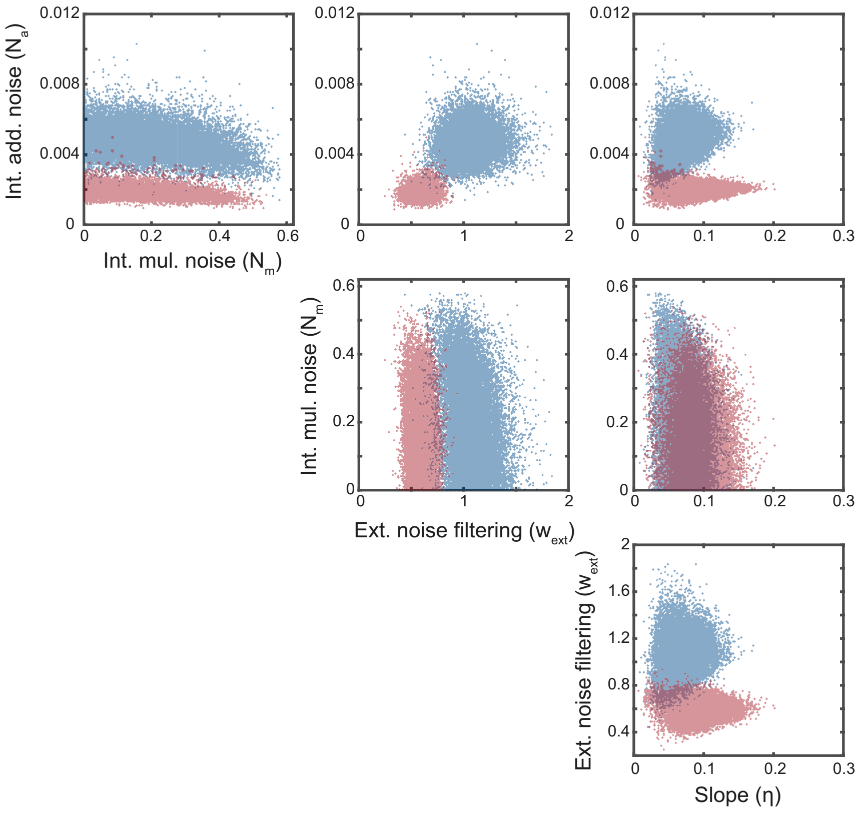

Figure S2. A bivariate correlation matrix plot for the model parameters in the hierarchical Bayesian PTM with fixed signal gain and nonlinearity. Blue data points represent samples from a participant with ASD, and red data points are from a participant with TD.

3. Convergence and autocorrelation in posterior samples

For the MCMC procedure, we ran 20 chains for each group, collecting every 200 samples after a burn-in of 15,000 samples, which resulted in 1,000 samples total in each chain. By running multiple chains, we were able to verify that the MCMC procedure converged to the regions with highest posterior probability. Figure S3 shows trace plots (first and third columns) and autocorrelation functions (second and fourth columns) for internal additive noise, internal multiplicative noise, external noise filtering, and slope of the psychometric function. Results from two chains are overlaid. The samples appear stationary (i.e., the mean does not seem to be changing across iterations), indicating that the MCMC procedure has converged. The results also show the absence of autocorrelations across samples.

To further confirm MCMC convergence, we calculated the potential scale reduction factor (PSRF[7]). This compares the variance within each chain and the variance between chains. In other words, PSRF should be close to 1. If the variance between chains is estimated to be substantially larger than the variance within a chain (> ~1.2), then this indicates lack of convergence. We used a multivariate version of PSRF[8]. The results confirmed convergence in MCMC samples for both groups (PSRF for ASD = 1.0042; for TD = 1.0045). Separate estimation of PSRF for each model parameter did not change the results.

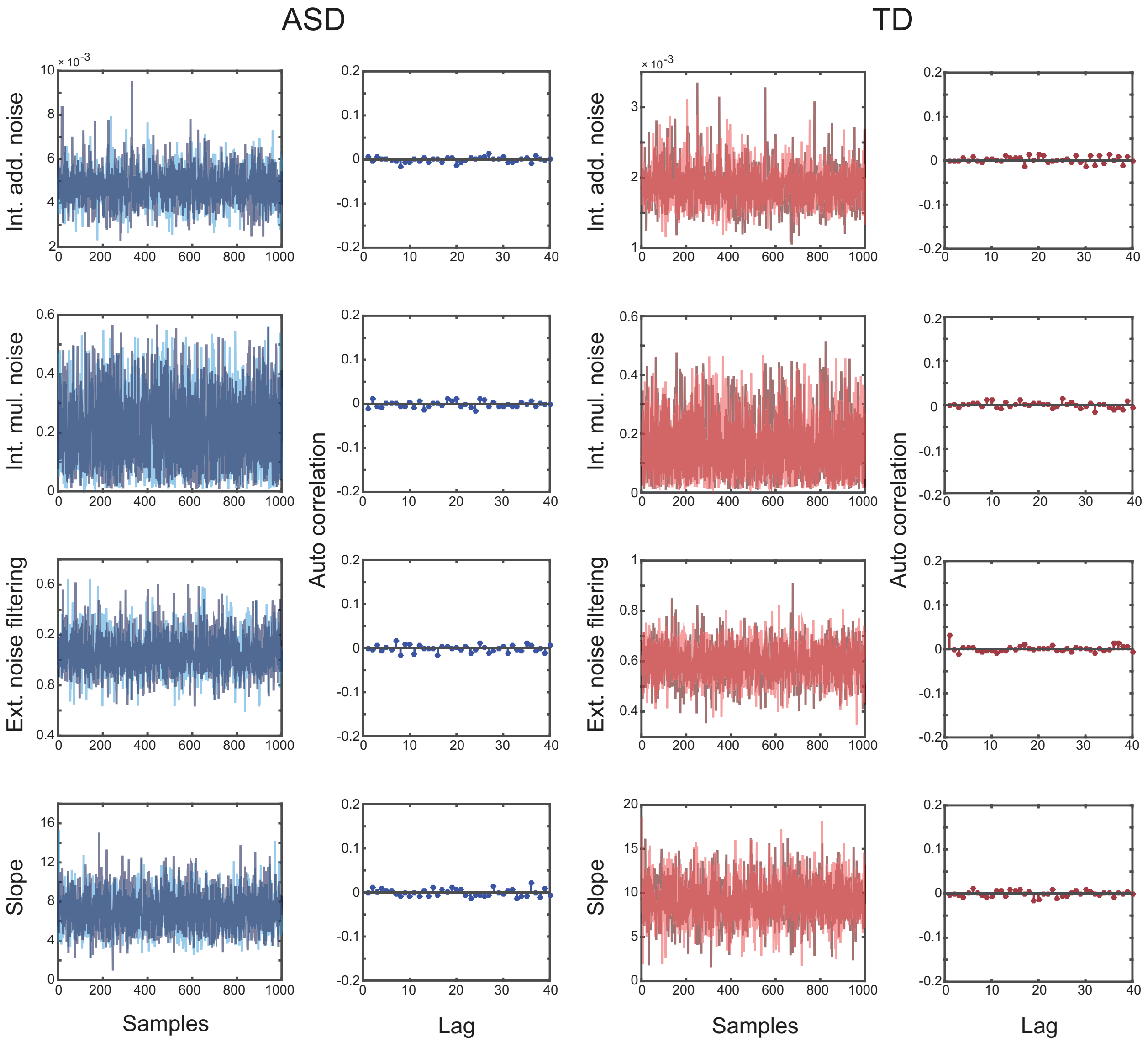

Figure S3. Trace plots (first and third columns) and autocorrelation functions (second and fourth columns) for the model parameter samples in the hierarchical Bayesian PTM obtained via the MCMC procedure. The first and second columns represent samples from a participant with ASD, and the third and fourth columns are from a participant with TD.

References

1. Dosher, B. A. & Lu, Z.-L. Mechanisms of perceptual learning. *Vision Res* **39,** 3197–3221 (1999).
2. Lu, Z.-L. & Dosher, B. A. Perceptual learning retunes the perceptual template in foveal orientation identification. *J Vis* **4,** 44–56 (2004).
3. Lu, Z.-L. & Dosher, B. A. External noise distinguishes attention mechanisms. *Vision Res* **38,** 1183–1198 (1998).
4. Lu, Z.-L. & Dosher, B. A. Characterizing human perceptual inefficiencies with equivalent internal noise. *J Opt Soc Am A Opt Image Sci Vis* **16,** 764–778 (1999).
5. Cavanaugh, M. R. *et al.* Visual recovery in cortical blindness is limited by high internal noise. *J Vis* **15,** 1–18 (2015).
6. Lu, Z.-L. & Dosher, B. A. Characterizing observers using external noise and observer models: assessing internal representations with external noise. *Psychol Rev* **115,** 44–82 (2008).
7. Gelman, A. & Rubin, D. B. Inference from iterative simulation using multiplie sequences. *Stat Sci* **7,** 457–511 (1992).
8. Brooks, S. P. & Gelman, A. General methods for monitoring convergence of iterative simulations. *J Comput Graph Stat* **7,** 434–455 (1998).
